# No Association Was Found Between Mild Iodine Deficiency During Pregnancy and Pregnancy Outcomes: a Follow-up Study Based on a Birth Registry

**DOI:** 10.1007/s12011-021-03028-y

**Published:** 2022-01-05

**Authors:** Xueying Cui, Huiting Yu, Zhengyuan Wang, Hai Wang, Zehuan Shi, Wei Jin, Qi Song, Changyi Guo, Hongmei Tang, Jiajie Zang

**Affiliations:** 1 Shanghai Municipal Center for Disease Control and Prevention, Shanghai, 200336 China; 2 Shanghai University of Traditional Chinese Medicine, Shanghai, 201203 China; 3 Minhang District Center for Disease Control and Prevention, Shanghai, 201101 China

**Keywords:** Mild iodine deficiency, Pregnancy, Outcomes, Urine iodine

## Abstract

**Background:**

Severe iodine deficiency during gestation is associated with adverse pregnancy outcomes; however, the impact of mild-to-moderate iodine deficiency, though prevalent in pregnancy, remains unclear.

**Methods:**

We extracted follow-up data for 7435 pregnant women from a national iodine deficiency disorders monitoring program from 2016 to 2018 and a mother–child cohort study in 2017 based on a birth registry in Shanghai. Birth outcomes were collected from the registry. Spot urine and household salt samples were collected for iodine testing. Single-factor analysis and logistic regression were used to evaluate the association between maternal iodine status and pregnancy outcomes.

**Results:**

The median urine iodine level in pregnant women was 137.5 μg/L (interquartile range 82.4–211.5), suggesting mild deficiency according to WHO standards. The incidence of pregnancy termination, preterm birth, congenital malformations, low birth weight, and cesarean section was 3.2%, 4.3%, 1.4%, 2.7%, and 45.2% in the mildly iodine-deficient group and 3.4%, 4.5%, 1.4%, 2.7%, and 44.5% in the normal group, respectively. After adjusting for maternal age and education, trimesters, and preterm birth rate in the general population, the odds ratios for any outcome did not differ significantly between the two groups.

**Conclusion:**

The present study suggests that mild maternal iodine deficiency is not associated with adverse pregnancy outcomes.

## Introduction

Iodine is an essential micronutrient for the human body. It is an important element in the synthesis of thyroid hormone, which is closely related to growth, nervous system development, and substance metabolism throughout the body [1]. Fetal demands for iodine must all come from the mother; thus, the iodine status of pregnant women has a direct influence on fetal growth and development [2]. The World Health Organization (WHO) recommends using the median urinary iodine concentration (UIC) to assess iodine status in populations. Based on the recommended criteria of iodine deficiency in pregnancy population by WHO [3] and other published criteria [4], iodine deficiency is defined as a median UIC < 150 μg/L (subcategorized as severe < 50 μg/L, moderate 50–99 μg/L, or mild100–149 μg/L). Populations are considered iodine sufficient with a median UIC of 150–249 μg/L, iodine excess at median UIC of 250–499 μg/L, or severe excess at median UIC of > 500 μg/L.

Since the implementation of the global salt iodization program in 1990, approximately 70% of the world’s households consume iodized salt. Universal salt iodization regulations were established in 1996 in China, with the standards for edible salt iodine content adjusted three times. As a result, the iodine intake of the general population is adequate [5], although mild iodine deficiency may be present in pregnant women [6–9].

Iodine deficiency during pregnancy can affect maternal thyroid function and pregnancy outcome as well as fetal growth and development. Severe iodine deficiency during pregnancy carries risks for the mother, including miscarriage, placental abruption, and stillbirth [10], as well as for the child, including cretinism, cognitive, and psychological defects, deafness, and thyroxin-related nerve damage [11–13]. However, the effects of mild-to-moderate iodine deficiency during pregnancy are controversial. Some studies have shown that it can lead to maternal and fetal thyroid dysfunction; however, it is not clear whether there is damage to the baby’s cognitive and neurological functions [14]. In contrast, a study found no association between mild-to-moderate maternal iodine deficiency and adverse pregnancy outcomes [15]. Daily iodine supplementation for mildly iodine-deficient pregnant women had no effect on neurodevelopment in children at 5–6 years of age [16]. An Indian study suggested that in areas where the general population has adequate iodine intake, mild-to-moderate maternal iodine deficiency has a limited impact on the offspring’s development [17]. However, some studies have found it to be associated with attention-deficit hyperactivity disorder [18] and cognitive dysfunction [19, 20] in children.

The increase in maternal blood volume during pregnancy may lead to a decrease in the concentration of nutrients, in turn resulting in a decline in urine concentration. Therefore, some have hypothesized that maternal iodine deficiency during pregnancy is a physiologic change, just like physiological anemia resulting from a decrease in hemoglobin concentration during pregnancy. In animal experiments, the placenta was found to be iodine-rich, and mild maternal iodine deficiency did no harm to the offspring [21–24]. Therefore, there is concern that oversupplementation with iodine during pregnancy may induce a state of excess iodine in some women, which could adversely affect fetal thyroid function, causing neonatal hypothyroidism [25–27].

Thus, the association between mild-to-moderate maternal iodine deficiency and pregnancy outcomes warrants further study. Over the past 4 years (2015–2018), the iodine status and pregnancy outcomes of women in Shanghai were monitored to investigate this question. In this study, we tested the association between urine iodine concentrations in pregnant women and pregnancy outcomes, including pregnancy termination, preterm birth, neonatal malformation, low birth weight, neonatal overweight, and a low Apgar score.

## Materials and Methods

### Study Design and Participants

This study included women in two cohort studies who had lived for over 1 year in the regions investigated and who had no recorded health problems including (1) heavy mental illness, hepatitis in the infected period, active tuberculosis, HIV infected, and other infectious diseases; (2) dementia and deaf-mute disabilities; (3) bedridden or activity limited; and (4) other diseases may disturb the study. All participants should comply with the inclusion and exclusion criteria and confirm written consent. The two studies were the national iodine deficiency disease (IDD) monitoring program from 2016 to 2018 and a cohort study evaluating the association between iodine status and offspring health in 2017. Both surveys were approved by the Medical Ethics Committee of Shanghai Municipal Center for Disease Prevention and Control and were aimed to continuously surveil participants’ iodine status via urine samples and family salt usage and include information on demography, lifestyle, medical history, and pregnancy as well as birth conditions in overall the same format. We aimed to detect the association between iodine status during pregnancy and gestational outcome. In each sampling unit, more than 20 pregnant women at different trimesters in each year were randomly recruited for a urine iodine test. At the same time, a household salt sample was collected from each woman’s home to test iodine content. A total of 4938 pregnant women were followed in the IDD monitoring program from 2016 to 2018. In the 2017 cohort study, complete follow-up data from 4805women and their offsprings were available.

### Sample Size

We calculated the sample size for logistic regression of a binary response variable (Y), which was mainly based on the following parameters: power, alpha (significance level), P_0_ (baseline probability that Y = 1), odds ratio (OR, Odds_1_/Odds_0_), and *R*-squared of X_1_ with other Xs. Preterm birth was set as the response variable (Y) and urine iodine status was set as the main independent variable (X). It was reported by the Shanghai Center for Women and Children’s Health that the preterm birth rate in general newborns of Shanghai was 5.53%(28) in 2016, which determined the value of P_0_. *R*-squared of X1 with other Xs was set at 0.0114 according to the results of the linear regression between maternal urine iodine status and potential confounders including maternal education, age, and trimesters. The power analysis for logistic regression was set for both-sided tests and main parameters including power, alpha, and OR which was set as 0.90, 0.05, and 0.7, respectively. According to the final results, the least sample size requires 1599 participants for the logistic regression analysis.

### Flowchart of the Study

**Fig. 1:**
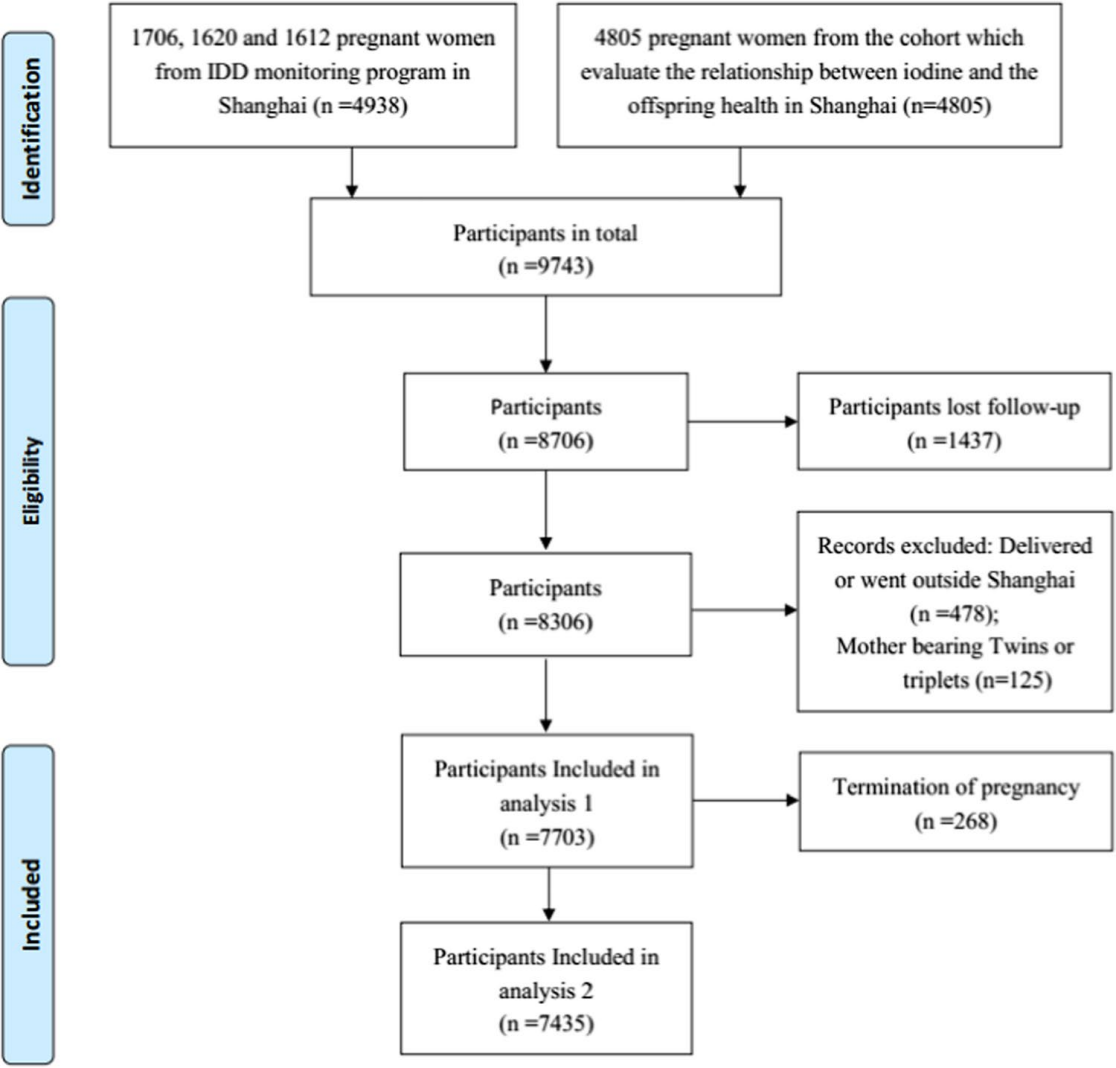
Flowchart of pregnant women in the study

Of the 9743 pregnant women eligible for the present study, we excluded those who were lost to follow-up or for whom information on UIC or neonatal outcome was incomplete (*n* = 1437), women who delivered outside of Shanghai (*n* = 478), those with twins or triplets (*n* = 125), or those who had pregnancy termination prior to study entry (*n* = 268). Finally, 7435 women with full information on iodine nutrition and pregnancy and neonatal outcomes were included in the analysis (Fig. 1).

### Evaluating Method and Criteria of Obstetric Outcomes

All women were face-to-face or telephonic interviewed during the pregnancy or after birth through the Shanghai birth registry, and then, details of obstetric outcome were extracted from hospital records following delivery. The mother’s age and education, whether the pregnancy was terminated, the mode of delivery, gestational weeks, congenital malformations, birth weight, birth length, and Apgar score were recorded.

The modes of delivery were reported as natural birth, cesarean delivery, forceps, fetal suction, and breech presentation. Preterm delivery was defined as birth before 37 completed weeks of gestation, whether by spontaneous onset of labor or iatrogenic delivery. Birth weight was categorized as low (< 2500 g), normal (2500–4000 g), or overweight (> 4000 g). The Apgar score is based on five physical signs in the neonate, including heart rate, respiration, muscle tone, laryngeal reflex, and skin color assessed at 1, 5, and 10 minutes after birth. The score was used to judge the presence and severity of neonatal asphyxia. An Apgar score of 8–10 points was defined as asphyxia free, 4–7 points as mild asphyxia, and 0–3 points as severe asphyxia. Because there were relatively few neonates with scores of 0–3 or 4–7, we combined those two groups and included a low Apgar score (< 8) in the logistic regression model. The incidence of each pregnancy outcome was analyzed according to UIC levels.

### Methods of Sampling and Testing and Evaluation Criteria of Urinary Iodine and Salt Iodine

The participants’ urine samples were collected at the community health service center on the day of the enrollment investigation. The participants discharge part of their urine first and then use a urine cup to collect mid-section urine for about 20 mL. The remaining urine can be discharged without collection, then transfer the urine sample from the urine cup to the urine collection tube, and tightly closed the tube cover. After collection, we temporarily store samples at 4 °C and – 80 °C for the long term. The iodine concentration of the spot urine sample was determined using the acid digestion method (As3 + -Ce4 + catalytic spectrophotometry) in the Shanghai Municipal Center for Disease Prevention and Control. Internal quality control samples for UIC were provided by the Chinese National Iodine Deficiency Disorders Reference Laboratory.

The iodine status of the pregnant women in the study was determined by the recommended WHO/UNICEF/ICCIDD criteria from 2007 [3] and other published criteria [17]. UIC of < 50 μg/L was considered severe insufficient: UIC of 50 to 100 μg/L moderate insufficient; UIC of 100 to150 mild insufficient; UIC of 150 to 249 μg/L adequate, UIC of > 250 μg/L greater than requirements or excessive.

Participants were required to take at least 20 g of the household salt sample using the light-proof sealed bag given by the investigators 3 days prior to the enrollment investigation. The iodine content of the household edible salt samples was determined by the direct titration method. According to the standard of edible salt iodine content of 30 mg/kg ± 30%, salt iodine content < 5 mg/kg was defined as non-iodized, 21 to 39 mg/kg as qualified or adequately iodized, and 5 to < 21 mg/kg or > 39 mg/kg as unqualified or inadequately iodized.

### Statistical Analysis

Continuous data are presented as median (interquartile range) and categorical data as number (percentage). UIC was not normally distributed, and differences between groups were therefore compared using a nonparametric test (Mann–Whitney *U* test). Additionally, since UICs were stratified into five groups as described above, chi-square tests and Fisher exact probability method were used when comparing UIC groups with the presence or absence of pregnancy outcomes. One-way analysis of variance was used to compare continuous data among the UIC groups. For data that were not normally distributed, the Kruskal–Wallis *H* test was used. Logistic regression was used to examine the association between maternal iodine status and the likelihood of adverse pregnancy outcomes, with the adequate UIC group as the reference.

Statistical significance was at the 0.05 level, and odds ratios (ORs) and 95% confidence intervals were calculated accordingly. All analyses were conducted using IBM SPSS Statistics version 21.0(IBM Corp., Armonk, NY, USA).

## Results

### Characteristic of the Study Population

The median age of the 7435 pregnant women was 29 (27 ~ 32) years. The median UIC was 137.5 (82.4–211.5) μg/L, with values of 137.3, 134.9, and 141.4 μg/L obtained in the first, second, or third trimester, respectively. These were all considered mildly deficient according to WHO standards. Iodized salt was found in 74.4% of the Shanghai households and qualified iodized salt in 60.1%. A total of 44.9% of the deliveries were by cesarean section, while 45.1% were vaginal, including natural, forceps, suction, or breech.

Among the 7435 women in the cohort, the median UIC was < 50 μg/L in 872 (11.7%), 50–99.9 μg/L in 1600 (21.5%), 100–149.9 μg/L in 1669 (22.5%), 150–249.9 μg/L in 1987 (26.7%), and > 250 μg/L in 1307 (17.6%) (Table 1).

**Table 1:** Demographic characteristics according to different levels of urinary iodine concentration (UIC) in pregnancy (*N*, %)

	All (*N*, %)	UIC (μg/L)	UIC groups (μg/L)				
			< 50	50–100	100–150	150–250	≥ 250
Mother age (years)							
Median (Q1–Q3)	29 (27 ~ 32)	-					
Maternal age							
< 25	771 (10.4)	139.8 (83.5 ~ 225.0)	90 (11.7)	154 (20.0)	173 (22.4)	204 (26.5)	150 (19.5)
25–30	3220 (43.3)	137.8 (84.2 ~ 209.0)	364 (11.3)	689 (21.4)	739 (23.0)	894 (27.8)	534 (16.6)
30–35	2406 (32.4)	138.0 (81.7 ~ 207.0)	291 (12.1)	507 (21.1)	540 (22.4)	653 (27.1)	415 (17.2)
≥ 35	1038 (14.0)	133.6 (76.6 ~ 219.0)	127 (12.2)	250 (24.1)	217 (20.9)	236 (22.7)	208 (20.0)
Mother education	7435						
Junior and below	907 (12.2)	149.4 (89.9 ~ 219.0)	106 (11.7)	168 (18.5)	181 (20.0)	292 (32.2)	160 (17.6)
High school	1296 (17.4)	140.1 (82.0 ~ 217.5)	156 (12.0)	263 (20.3)	292 (22.5)	351 (27.1)	234 (18.1)
College and above	5232 (70.4)	135.0 (81.1 ~ 208.0)	610 (11.7)	1169 (22.3)	1196 (22.9)	1344 (25.7)	913 (17.5)
Mother obesity	7435						
Body mass index	21.7 ± 6.3	-	21.4 ± 5.6*	21.5 ± 3.3	21.6 ± 5.0	21.8 ± 4.4*	21.9 ± 10.5
Obesity^#1^	244	149.3 (86.9 ~ 231.1)	24 (2.7)	46 (2.9)	52 (3.5)	69 (3.6)	53 (3.3)
Pre-diagnosed obesity	140	149.4 (87.8 ~ 224.4)	12 (1.4)	34 (0.8)	25 (0.8)	42 (0.6)	27 (0.7)
Trimester	7435						
1-trimester	2852 (38.4)	137.3 (84.4 ~ 212.0)	324 (11.4)	605 (21.2)	640 (22.4)	769 (27.0)	514 (18.0)
2-trimester	2747 (36.9)	134.9 (78.9 ~ 210.0)	334 (12.2)	623 (22.7)	610 (22.2)	702 (25.6)	478 (17.4)
3-trimester	1836 (24.7)	141.4 (84.9 ~ 213.5)	214 (11.7)	372 (20.3)	419 (22.8)	516 (28.1)	315 (17.2)
Iodized salt	6237						
Qualified iodized salt	3748 (60.1)	143.0 (83.0 ~ 223.4)	437 (11.7)	778 (20.8)	771 (20.6)	999 (26.6)	763 (20.4)
Non-iodized salt	1605 (25.7)	126.0 (74.8 ~ 203.0)	229 (14.3)	374 (23.3)	356 (22.2)	386 (24.0)	260 (16.2)
Iodized salt	4632 (74.3)	140.1 (81.4 ~ 220.4)	555 (12.0)	992 (21.4)	957 (20.7)	1225 (26.4)	903 (19.5)
Mode of delivery	7435						
Natural birth	3923 (52.76)	136.3 (81.1 ~ 210.0)	488 (56.0)	825 (51.6)	885 (53.0)	1050 (52.8)	675 (51.6)
Cesarean delivery	3339 (44.91)	139.4 (83.6 ~ 214.0)	360 (41.3)	736 (46)	754 (45.2)	885 (44.5)	604 (46.2)
Forceps delivery	163 (2.19)	134.2 (83.3 ~ 207.0)	21 (2.4)	37 (2.3)	29 (1.7)	49 (2.5)	27 (2.1)
Vacuum extraction	8 (0.11)	98.8 (41.9 ~ 234.0)	2 (0.2)	2 (0.1)	1 (0.1)	2 (0.1)	1 (0.1)
Breech delivery	2 (0.03)	114.2 (42.3 ~)	1 (0.1)	0 (0)	0 (0)	1 (0.1)	0 (0)
Pregnancy history	7435						
Pregnancy loss^#2^	677 (0.09)	145.1 (87.6 ~ 227.8)	73 (8.2)	132 (8.4)	139 (9.5)	172 (9.0)	161 (10.0)
Early birth	108 (0.15)	135.3 (77.45 ~ 212.6)	11 (1.1)	22 (1.4)	23 (1.6)	33 (1.7)	19 (1.3)
Low birth weight	71 (0.01)	152.2 (62.2 ~ 216.6)	11 (1.1)	17 (1.1)	6 (0.4)	21 (1.1)	16 (1.1)
Total	7435	137.5 (82.4 ~ 211.5)	872 (11.7)	1600 (21.5)	1669 (22.5)	1987 (26.7)	1307 (17.6)

^#1^Obesity definition was according to the mothers’ body mass index (BMI) before pregnancy

^#2^Pregnancy loss includes abortion, miscarriage, and stillbirth

^*^Statistical difference between two groups

### Obstetric Outcomes

During the study period, 3.5% of the pregnant women underwent pregnancy termination. The incidence of preterm birth was 4.1%, and cesarean deliveries were performed in 44.9%. Among the neonates, 1.3% showed congenital malformations, 2.4% were underweight, and 5.9% were overweight. Among full-term infants, 0.8% showed low birthweight, which was significantly lower than that in the entire cohort. Apgar scores at 1, 5, and 10 min, respectively, were 1–3 in 0.11%, 0.03%, and 0.02%; 4–7 in 0.99%, 0.13%, and 0.11%; and 8–10 in 98.90%, 99.84%, and 99.87%, respectively (Table 2). Among the full-term newborns, the median weight was 3350.0 (3075.0–3620.0) g and median length 50.0 (50.0–50.0) cm (Table 3).

**Table 2:** Comparison of adverse pregnancy outcomes according to maternal iodine status

	Age (years)	*N*	*n* (%)	< 50		50–100		100–150		150–250		≥ 250		*χ*^*2*^	*P* value
				*N*	*n* (%)	*N*	*n* (%)	*N*	*n* (%)	*N*	*n* (%)	*N*	*n* (%)		
Termination of pregnancy	< 25	819	48 (5.8)	96	6 (6.3)	164	10 (6.1)	183	10 (5.5)	217	13 (6.0)	159	9 (5.7)	0.069	0.999
	25–30	3302	82 (2.4)	373	9 (2.4)	707	18 (2.5)	756	17 (2.2)	914	20 (2.2)	552	18 (3.3)	1.898	0.756
	30–35	2506	90 (3.6)	300	9 (3.0)	525	18 (3.4)	560	20 (3.6)	678	25 (3.7)	433	18 (4.2)	0.758	0.944
	≥ 35	1076	48 (4.4)	133	6 (4.5)	261	11 (4.2)	226	9 (4.0)	248	12 (4.8)	218	10 (4.0)	1.652	0.799
	All	7703	268 (3.5)	902	30 (3.3)	1657	57 (3.4)	1725	56 (3.2)	2057	70 (3.4)	1362	55 (4.0)	28.141	0.081
Preterm birth	< 25	771	23 (3.0)	90	2 (2.2)	154	6 (3.9)	173	4 (2.3)	204	9 (4.4)	150	2 (1.3)	3.742	0.442
	25–30	3220	127 (3.9)	364	11 (3.0)	689	23 (3.3)	739	34 (4.6)	894	42 (4.7)	534	17 (3.2)	4.482	0.345
	30–35	2406	95 (3.9)	291	14 (4.8)	507	17 (3.4)	540	25 (4.6)	653	24 (3.7)	415	15 (3.6)	1.956	0.744
	≥ 35	1038	58 (5.6)	127	5 (3.9)	250	20 (8.0)	217	8 (3.7)	236	15 (6.4)	208	10 (4.8)	5.404	0.248
	All	7435	303 (4.1)	872	32 (3.7)	1600	66 (4.1)	1669	71 (4.3)	1987	90 (4.5)	1307	44 (3.4)	3.241	0.518
Congenital malformations	< 25	771	14 (1.8)	90	1 (1.1)	154	2 (1.3)	173	3 (1.7)	204	5 (2.5)	150	3 (2.0)	0.978	0.913
	25–30	3220	38 (1.2)	364	4 (1.1)	689	10 (1.5)	739	9 (1.2)	894	10 (1.1)	534	5 (0.9)	0.766	0.943
	30–35	2406	29 (1.2)	291	4 (1.4)	507	7 (1.4)	540	8 (1.5)	653	8 (1.2)	415	2 (0.5)	2.373	0.668
	≥ 35	1038	15 (1.4)	127	1 (0.8)	250	4 (1.6)	217	4 (1.8)	236	5 (2.1)	208	1 (0.5)	2.779	0.595
	All	7435	96 (1.3)	872	10 (1.1)	1600	23 (1.4)	1669	24 (1.4)	1987	28 (1.4)	1307	11 (0.8)	2.983	0.561
Low birth weight	< 25	771	16 (2.1)	90	1 (1.1)	154	4 (2.6)	173	3 (1.7)	204	5 (2.5)	150	3 (2.0)	0.793	0.939
	25–30	3220	73 (2.3)	364	5 (1.4)	689	11 (1.6)	739	21 (2.8)	894	25 (2.8)	534	11 (2.1)	5.169c	0.270
	30–35	2406	67 (2.8)	291	8 (2.7)	507	11 (2.2)	540	16 (3.0)	653	18 (2.8)	415	14 (3.4)	1.315d	0.859
	≥ 35	1038	26 (2.5)	127	2 (1.6)	250	9 (3.6)	217	5 (2.3)	236	6 (2.5)	208	4 (1.9)	1.884e	0.757
	All	7435	182 (2.4)	872	16 (1.8)	1600	35 (2.2)	1669	45 (2.7)	1987	54 (2.7)	1307	32 (2.4)	2.927a	0.570
Low birth weight of full-month babies	< 25	748	6 (0.8)	88	0	148	1 (0.7)	169	0	195	3 (1.5)	148	2 (1.4)	3.937	0.415
	25–30	3093	20 (0.6)	353	2(0.6)	666	4(0.6)	705	6(0.9)	852	4(0.5)	517	4(0.8)	1.123	0.891
	30–35	2311	24 (1.0)	277	2 (0.7)	490	4 (0.8)	515	5 (1.0)	629	8 (1.3)	400	5 (1.3)	0.998	0.910
	≥ 35	980	7 (0.7)	122	1 (0.8)	230	3 (1.3)	209	0	221	2 (0.9)	198	1 (0.5)	2.815	0.589
	All	7132	57 (0.8)	840	5 (0.6)	1534	12 (0.8)	1598	11 (0.7)	1897	17 (0.9)	1263	12 (1.0)	1.269	0.867
Birth overweight	< 25	771	41 (5.3)	90	7 (7.8)	154	7 (4.5)	173	10 (5.8)	204	9 (4.4)	150	8 (5.3)	1.585	0.812
	25–30	3220	175 (5.4)	364	19 (5.2)	689	30 (4.4)	739	45 (6.1)	894	46 (5.1)	534	35 (6.6)	3.791	0.435
	30–35	2406	148 (6.2)	291	19 (6.5)	507	30 (5.9)	540	39 (7.2)	653	37 (5.7)	415	23 (5.5)	1.742	0.783
	≥ 35	1038	74 (7.1)	127	8 (6.3)	250	15 (6.0)	217	16 (7.4)	236	15 (6.4)	208	20 (9.6)	2.673	0.614
	All	7435	438 (5.9)	872	53 (6.1)	1600	82 (5.1)	1669	110 (6.6)	1987	107 (5.4)	1307	86 (6.6)	5.325	0.256
Cesarean delivery	< 25	771	272 (35.3)	90	26 (28.9)	154	63 (40.9)	173	65 (37.6)	204	64 (31.4)	150	54 (36.0)	20.945	0.051
	25–30	3220	1267 (39.3)	364	133 (36.5)	689	273 (39.6)	739	287 (38.8)	894	352 (39.4)	534	222 (41.6)	11.864	0.753
	30–35	2406	1180 (49.0)	291	128 (44.0)	507	252 (49.7)	540	266 (49.3)	653	337 (51.6)	415	197 (47.5)	16.898	0.392
	≥ 35	1038	620 (59.7)	127	73 (57.5)	250	148 (59.2)	217	136 (62.7)	236	132 (55.9)	208	131 (63.0)	6.627	0.577
	All	7435	3339 (44.9)	872	360 (41.3)	1600	736 (46.0)	1669	754 (45.2)	1987	885 (44.5)	1307	604 (46.2)	14.519	0.560
Apgar 1	1–3		8 (0.1)		1 (0.1)		2 (0.1)		1 (0.1)		3 (0.2)		1 (0.1)	6.111	0.635
	4–6		72 (1.0)		7 (0.8)		17 (1.1)		9 (0.5)		23 (1.2)		16 (1.2)		
	7–10		7355 (98.9)		864 (99.1)		1581 (98.8)		1659 (99.4)		1961 (98.7)		1290 (98.7)		
Apgar 5	1–3		2 (0)		0 (0.0)		0 (0.0)		0 (0.0)		2 (0.1)		0 (0.0)	9.429	0.307
	4–6		10 (0.1)		0 (0.0)		2 (0.1)		1 (0.1)		5 (0.3)		2 (0.2)		
	7–10		7423 (99.8)		872 (100.0)		1598 (99.9)		1668 (99.9)		1980 (99.6)		1305 (99.8)		
Apgar 10	1–3		1 (0.0)		0 (0.0)		0 (0.0)		0 (0.0)		1 (0.1)		0 (0.0)	5.296	0.726
	4–6		6 (0.1)		0 (0.0)		2 (0.2)		2 (0.2)		2 (0.1)		0 (0.0)		
	7–10		5501 (99.9)		628 (100.0)		1183 (99.8)		1273 (99.8)		1451 (99.8)		966 (100.0)

**Table 3:** Comparison of weight and length among full-term babies according to different levels of maternal urinary iodine concentrations

	Age (years)	Median (Q1–Q3)	< 50	50–100	100–150	150–250	≥ 250	*χ*^2^	*P* value
Birth weight	< 25	3300.0 (3050.0 ~ 3600.0)	3300.0 (3060.0 ~ 3657.5)	3322.5 (3100.0 ~ 3600.0)	3350.0 (3100.0 ~ 3600.0)	3260.0 (3030.0 ~ 3600.0)	3355.0 (3150.0 ~ 3656.3)	4.341	0.362
	25–30	3330.0 (3060.0 ~ 3601.3)	3320.0 (3080.0 ~ 3590.0)	3350.0 (3100.0 ~ 3600.0)	3375.0 (3100.0 ~ 3660.0)	3350.0 (3095.0 ~ 3610.0)	3340.0 (3087.5 ~ 3630.0)	3.561	0.469
	30–35	3360.0 (3091.3 ~ 3640.0)	3350.0 (3125.0 ~ 3630.0)	3385.0 (3136.3 ~ 3660.0)	3400.0 (3150.0 ~ 3660.0)	3380.0 (3105.0 ~ 3640.0)	3387.5 (3100.0 ~ 3653.8)	2.062	0.724
	≥ 35	3350.0 (3096.3 ~ 3640.0)	3305.0 (3153.8 ~ 3550.0)	3400.0 (3123.8 ~ 3650.0)	3380.0 (3150.0 ~ 3682.5)	3350.0 (3095.0 ~ 3647.5)	3400.0 (3145.0 ~ 3725.0)	4.084	0.395
	All	3350.0 (3075.0 ~ 3620.0)	3332.5 (3105.0 ~ 3600.0)	3365.0 (3100.0 ~ 3635.0)	3380.0 (3120.0 ~ 3650.0)	3350.0 (3095.0 ~ 3630.0)	3360.0 (3100.0 ~ 3650.0)	7.258	0.123
Birth length	< 25	50.0 (50.0 ~ 50.0)	50.0 (50.0 ~ 50.0)	50.0 (50.0 ~ 50.0)	50.0 (50.0 ~ 50.0)	50.0 (50.0 ~ 50.0)	50.0 (50.0 ~ 50.0)	5.660	0.226
	25–30	50.0 (50.0 ~ 50.0)	50.0 (50.0 ~ 50.0)	50.0 (50.0 ~ 50.0)	50.0 (50.0 ~ 50.0)	50.0 (50.0 ~ 50.0)	50.0 (50.0 ~ 50.0)	3.881	0.422
	30–35	50.0 (50.0 ~ 50.0)	50.0 (50.0 ~ 50.0)	50.0 (50.0 ~ 50.0)	50.0 (50.0 ~ 50.0)	50.0 (50.0 ~ 50.0)	50.0 (50.0 ~ 50.0)	2.972	0.563
	≥ 35	50.0 (50.0 ~ 50.0)	50.0 (50.0 ~ 50.0)	50.0 (50.0 ~ 50.0)	50.0 (50.0 ~ 50.0)	50.0 (50.0 ~ 50.0)	50.0 (50.0 ~ 50.0)	0.707	0.950
	All	50.0 (50.0 ~ 50.0)	50.0 (50.0 ~ 50.0)	50.0 (50.0 ~ 50.0)	50.0 (50.0 ~ 50.0)	50.0 (50.0 ~ 50.0)	50.0 (50.0 ~ 50.0)	2.695	0.610

### Adverse Pregnancy Outcomes According to Maternal Urinary Iodine

The pregnant women were divided into four age groups, and then, the incidence of adverse obstetric outcomes in each group was compared according to five UIC levels. There were no significant differences among the five UIC groups in terms of Apgar scores or the incidence of preterm births, cesarean deliveries, congenital malformations, underweight, or overweight (Table 2).

To exclude the influence of preterm birth, 7312 cases of full-term birth were reanalyzed based on the mothers’ age groups. The low birth weight incidence and the newborns’ weights and lengths were compared according to UIC levels. Again, none of the groups differed significantly (Tables 2 and 3).

### Logistic Regression Analysis

We calculated ORs of adverse pregnancy outcomes including preterm birth, congenital malformations, low birthweight, overweight, cesarean delivery, and low Apgar scores among the UIC groups with models adjusting for the confounders of mother’s age and education and the trimester when the urine samples were collected. When assessing low birthweight, the model was also adjusted for preterm birth. None of the adjusted ORs differed significantly for any of the adverse outcomes (Table 4).

**Table 4:** Multivariate analysis of adverse pregnancy outcomes in women with different urine iodine concentrations

Pregnancy outcomes	UIC Groups (μg/L)	Adjusted OR1*		Adjusted OR2**	
		OR (95% CI)	*P* value	AOR (95% CI)	*P* value
Preterm birth	< 50	0.8 (0.5–1.2)	0.30	0.8 (0.5–1.2)	0.28
	50–100	0.9 (0.7–1.3)	0.56	0.9 (0.7–1.3)	0.53
	100–150	0.9 (0.7–1.3)	0.69	0.9 (0.7–1.3)	0.70
	150–250	1.0	0.52	1.0	0.48
	≥ 250	0.7 (0.5–1.1)	0.10	0.7 (0.5–1.1)	0.09
Congenital malformations	< 50	0.8 (0.4–1.7)	0.57	0.8 (0.4–1.7)	0.57
	50–100	1.0 (0.6–1.8)	0.94	1.0 (0.6–1.8)	0.95
	100–150	1.0 (0.6–1.8)	0.94	1.0 (0.6–1.8)	0.93
	150–250	1.0	0.57	1.0	0.57
	≥ 250	0.6 (0.3–1.2)	0.15	0.6 (0.3–1.2)	0.14
Low birth weight**	< 50	0.7 (0.4–1.2)	0.17	0.7 (0.4–1.3)	0.28
	50–100	0.8 (0.5–1.2)	0.30	0.8 (0.5–1.3)	0.35
	100–150	1.0 (0.7–1.5)	0.98	1.1 (0.7–1.7)	0.85
	150–250	1.0	0.57	1.0	0.54
	≥ 250	0.9 (0.6–1.4)	0.68	1.1 (0.7–1.9)	0.64
Birth overweight	< 50	1.1 (0.8–1.6)	0.49	1.1 (0.8–1.6)	0.47
	50–100	0.9 (0.7–1.3)	0.70	1.0 (0.7–1.3)	0.77
	100–150	1.2 (0.9–1.6)	0.13	1.3 (1.0–1.7)	0.10
	150–250	1.0	0.26	1.0	0.25
	≥ 250	1.2 (0.9–1.7)	0.16	1.2 (0.9–1.7)	0.15
Cesarean delivery	< 50	0.9 (0.8–1.0)	0.11	0.9 (0.7–1.0)	0.07
	50–100	1.1 (0.9–1.2)	0.38	1.1 (0.9–1.2)	0.45
	100–150	1.0 (0.9–1.2)	0.70	1.0 (0.9–1.2)	0.59
	150–250	1.0	0.17	1.0	0.14
	≥ 250	1.1 (0.9–1.2)	0.35	1.1 (0.9–1.2)	0.44
Low Apgar 1 score	< 50	0.7 (0.3–1.6)	0.38	0.7 (0.3–1.6)	0.42
	50–100	0.9 (0.5–1.6)	0.75	1.0 (0.5–1.7)	0.88
	100–150	0.5 (0.2–1.0)	0.035	0.5 (0.2–1.0)	0.045
	150–250	1.0	0.25	1.0	0.28
	≥ 250	1.0 (0.5–1.8)	0.98	1.0 (0.6–1.9)	0.96
Low Apgar 5 score	< 50	0.0 (0.0–0.0)	0.99	0.0 (0.0–0.0)	0.99
	50–100	0.4 (0.1–1.7)	0.20	0.4 (0.1–1.8)	0.23
	100–150	0.2 (0.0–1.4)	0.10	0.2 (0.0–1.5)	0.11
	150–250	1.0	0.39	1.0	0.43
	≥ 250	0.4 (0.1–2.1)	0.30	0.4 (0.1–2.1)	0.30
Low Apgar 10 score	< 50	0.0(0.0–0.0)	0.99	0.0 (0.0–0.0)	0.99
	50–100	0.8(0.1–4.9)	0.83	0.8 (0.1–5.1)	0.85
	100–150	0.8(0.1–4.6)	0.76	0.8 (0.1–4.7)	0.79
	150–250	1.0	1.00	1.0	1.00
	≥ 250	0.00 (0.0–0.0)	0.99	0.00 (0.0–0.0)	0.99

^*^Logistic regression model adjusted for mother’s age and education and trimester of urine samples

^**^Logistic regression model adjusted for mother’s age and education, trimester of urine samples, and preterm births in a general population in Shanghai. The reference group was women with a normal urine iodine concentration (150–249 μg/L)

## Discussion

In this study, we found that mild iodine deficiency in pregnant women was not associated with an adverse pregnancy outcome including pregnancy termination, preterm birth, congenital malformations, low birth weight, or overweight.

Because of increased thyroid hormone synthesis, transfer of iodine to the fetus, and increased glomerular filtration rates resulting in increased passive loss of iodine in the urine, pregnant women require a higher iodine intake than non-pregnant women [29, 30]. We found that many pregnant women in Shanghai were in the state of mild iodine deficiency according to the current WHO standard. However, we did not find any adverse maternal or neonatal outcomes associated with this deficiency. According to the published incidence of congenital hypothyroidism (CH) in newborns in Shanghai, it has been a relatively low level at 32.50 per 100,000 people from 1997 to 2007 [31, 32], which is lower than that in China at 49.2 per 100,000 people from 1985 to 2006 [33]. This is consistent with our findings, supporting our contention that mild gestational iodine deficiency does no harm to mothers or their newborns. Another study reported that daily iodine supplementation in women with mild iodine deficiency had no effect on their children’s neurodevelopment assessed at the age of 5 or 6 years [18]. Therefore, iodine supplements should not automatically be prescribed for pregnant women just because the population is mildly iodine deficient, particularly when the safe upper limit of iodine intake for pregnant women is unknown.

Both iodine deficiency and excess have been posited to be associated with adverse effects on thyroid function since iodine is an essential constituent of thyroid hormones [34]. However, most individuals reportedly tolerate high exposure to iodine or compensate for mild-to-moderate iodine deficiency by means of homeostatic mechanisms [35]. There is evidence that maintaining an adequate iodide supply for the developing fetus is dependent on both maternal dietary iodine intake and placental iodide transport. Pregnancy-associated hormones, particularly oxytocin and human chorionic gonadotropin, help promote placental iodide uptake, which may protect the fetus against iodine deficiency [24]. The results of our study show that mild maternal iodine deficiency is not associated with adverse pregnancy outcomes, suggesting that unlike the thyroid and brain, maternal and fetal placental tissues are not particularly sensitive to minor perturbations in iodine availability and can therefore continue to function normally in pregnancy. The effects of iodine deficiency might also be compensated for by the population-wide improvement in nutritional status as living standards have improved.

We found no association between different maternal UICs and pregnancy outcomes, consistent with a British study that also found no significant difference in the incidence of preterm delivery and mode of delivery among groups based on the urinary iodine-to-creatinine ratio [15]. A study in Spain demonstrates that birthweight was associated with maternal iodine status. They found neonates born to women with a third trimester UIC between 100 μg/L and 149 μg/L were less likely to be small for gestational age than those born to women with a UIC of below 50 μg/L [36]. This study provides side proof to the minor severity of health risks due to mild iodine deficiency and also hints that severe iodine deficiency may be more likely to be associated with adverse birth outcomes than mild or moderate iodine deficiency. However, our findings contradict those of a study in Thailand in which the rates of preterm birth and low birthweight were significantly higher in women with iodine deficiency. They found that iodine status was an independent risk factor for preterm birth and low birthweight [37]. Given these conflicting results, the true association between the iodine status of pregnant women and neonatal outcomes requires further study.

Some recent studies have suggested that mild maternal iodine deficiency may be associated with a low IQ, poor language development [38–40], or attention-deficit hyperactivity disorder [41] in their children. Another study has reported that iodine deficiency might adversely affect women’s fertility [42]. The reasons for these discrepancies are unclear. However, different confounders related to iodine deficiency may affect pregnancy outcomes in different settings.

The main advantages of our study are the robust evidence derived from a large sample size of pregnant women and their newborns that were followed up in the IDD monitoring program, which supports the strength of our findings. However, one limitation of this study is the limited number of covariates considered. In addition, the use of only one urinary iodine measurement to classify iodine status may not represent a woman’s iodine status throughout pregnancy [43]. Therefore, there may have been some misclassification, which could mask possible differences between groups. Besides, because the incidence of some adverse pregnancy outcomes in this study was very low, the study may have been underpowered to detect differences for those outcomes. At last, Shanghai is an environmentally iodine-deficient area, but due to the sufficient intake of iodine-rich seafood as well as the iodized salt, and the high overall dietary quality of the population, the overall iodine level of the population in Shanghai is appropriate. Therefore, mild to moderate iodine deficiency of pregnant women in Shanghai may have a limited impact on pregnancy and birth outcomes. However, in other areas where iodine is deficient in the environment and the population’s overall intake of iodine and dietary quality is insufficient or in certain areas with high iodine level in the environment, the study’s results may be less applicable.

## Conclusions

Mild maternal iodine deficiency is not associated with adverse pregnancy outcomes and may not be a threat to pregnancy outcomes.

## Data Availability

Restrictions apply to the availability of data generated or analyzed during this study to preserve patient confidentiality or because they were used under license. The corresponding author will on request detail the restrictions and any conditions under which access to some data may be provided.
